# Effect of Preconception Selenium Intake on the Risk for Gestational Diabetes: The Japan Environment and Children’s Study

**DOI:** 10.3390/antiox10040568

**Published:** 2021-04-07

**Authors:** Hyo Kyozuka, Tsuyoshi Murata, Toma Fukuda, Akiko Yamaguchi, Aya Kanno, Shun Yasuda, Akiko Sato, Yuka Ogata, Mitsuaki Hosoya, Seiji Yasumura, Koichi Hashimoto, Hidekazu Nishigori, Keiya Fujimori

**Affiliations:** 1 Fukushima Regional Center for the Japan Environmental and Children’s Study, 1 Hikarigaoka, Fukushima 960-1295, Japan; tuyoshim@fmu.ac.jp (T.M.); t323@fmu.ac.jp (T.F.); akiko-y@fmu.ac.jp (A.Y.); ayao0827@fmu.ac.jp (A.K.); room335@fmu.ac.jp (S.Y.); asato@fmu.ac.jp (A.S.); yuka-o@fmu.ac.jp (Y.O.); mhosoya@fmu.ac.jp (M.H.); yasumura@fmu.ac.jp (S.Y.); don@fmu.ac.jp (K.H.); nishigo@fmu.ac.jp (H.N.); fujimori@fmu.ac.jp (K.F.); 2 Department of Obstetrics and Gynecology, Fukushima Medical University School of Medicine, 1 Hikarigaoka, Fukushima 960-1295, Japan; 3 Department of Pediatrics, Fukushima Medical University School of Medicine, 1 Hikarigaoka, Fukushima 960-1295, Japan; 4 Department of Public Health, Fukushima Medical University School of Medicine, 1 Hikarigaoka, Fukushima 960-1295, Japan; 5 Fukushima Medical Center for Children and Women, Fukushima Medical University, 1 Hikarigaoka, Fukushima 960-1295, Japan

**Keywords:** cohort studies, dietary selenium, preconception care, gestational diabetes

## Abstract

Selenium (Se) acts as a cofactor of antioxidant enzymes. Preconception care may reduce the risk of gestational diabetes mellitus (GDM). We examined the association between preconception Se intake and the risk of GDM in Japanese women. Using the Japan Environment and Children’s Study database, we identified 92,764 Japanese women recruited between January 2011 and March 2014. Participants were categorized into five groups according to preconception Se intake quintiles (Q1 and Q5 were the lowest and highest Se intake groups, respectively). GDM was categorized as early-onset (Eo-GDM) or late-onset (Lo-GDM) diagnosed before or after 24 weeks, respectively. Multiple logistic regressions were performed to identify the effect of preconception Se intake on GDM, Eo-GDM, and Lo-GDM. Using Q3 (the middle Se intake group) as the reference, a multiple logistic regression analysis showed that the highest (Q5) Se intake group demonstrated increased risks of GDM (adjusted odds ratio (aOR): 1.15, 95% confidence interval (CI): 1.01–1.30) and the lowest (Q1) Se intake group had increased risks of Lo-GDM (aOR: 1.19, 95% CI: 1.01–1.41). Thus, both high and low preconception Se intakes increase risks of glucose intolerance during pregnancy. This finding may indicate new recommendations for preconception Se intake to prevent GDM.

## 1. Introduction

Gestational diabetes mellitus (GDM) is a condition of carbohydrate intolerance that occurs during pregnancy [1]. GDM has long-term effects on the health of both the mother and child. Approximately 70% of women with GDM develop diabetes mellitus (DM) within 22–28 years after pregnancy [2]. GDM also increases the offspring’s risk for developing obesity, impaired glucose tolerance, and DM [3,4]. Poorly controlled maternal GDM may also cause poor neurodevelopmental outcomes [5]. A major challenge in modern obstetrics—and one of the major public health concerns—is the development of preconception care (in the form of counseling or interventions) to reduce the prevalence of GDM. These interventions seek to change dietary habits before pregnancy to help maintain the health of the mother and child in the long term [6]. 

Selenium (Se) is a micronutrient that acts as a cofactor of antioxidant enzymes, which counterbalance oxidative stress and regulate the inflammatory response [7,8]. Se is integrated into proteins to make selenoproteins (SP). In addition to its antioxidant effect, excessive SP also impairs glucose intolerance and insulin resistance in type 2 diabetes [9,10] 

The placenta is the main source of reactive oxygen species (ROS), which induce cellular damage by reacting with proteins, lipids, and nucleic acids [11]. Under normal physiological conditions, endogenous oxidants and various antioxidant systems are balanced. Excessive production of ROS or impaired antioxidant defense can produce several pregnancy complications, such as pre-eclampsia and preterm birth, which affect not only maternal morbidity but also both maternal and neonate future health [12,13,14]. However, Se deficiency, which reduces antioxidant defenses, may also be a potential risk for GDM, owing to oxidative stress [15]. Oxidative stress can impair glucose tolerance and insulin resistance and induce systemic endothelial dysfunction. These factors may directly or indirectly contribute to impaired pancreatic beta-cell function and glucose intolerance [16].

Previous studies reported that reduced maternal Se levels are associated with recurrent early pregnancy loss and pre-eclampsia [17,18,19,20,21]. However, there is little evidence regarding the effect of preconception Se intake on the development of GDM via its antioxidant effects. One of the oxidative DNA damage products, 8-hydroxy-2′-deoxyguanosine (8-OHdG), is excreted directly into the urine and is a sensitive biomarker of oxidative stress [22]. In this study, we examined whether maternal preconception Se intake was associated with the occurrence of GDM in Japanese women using data from a large Japanese cohort study. We used the maternal urine 8-OHdG concentrations during pregnancy to assess maternal oxidative stress.

## 2. Materials and Methods 

### 2.1. Study Design

The present study included an analysis of data from the Japan Environment and Children’s Study (JECS), a government-funded birth cohort study that started in January 2011 and investigated the effects of several environmental factors on children’s health [23]. Pregnant women were eligible for JECS participation if they met the following criteria: (1) lived in the study area at the time of the application and expected to live in Japan in the near future; (2) had an expected delivery date between 1 August 2011, and mid-2014; and (3) were able to participate without difficulty (i.e., they could complete the self-management questionnaires). 

The JECS protocol was reviewed and approved by the Institutional Review Board (approved 10 September S2010) on Epidemiological Studies of the Ministry of the Environment and by the ethics committees of all participating institutions. The JECS was conducted in accordance with the Helsinki Declaration and other nationally valid regulations and guidelines. Written informed consent was obtained from all participants.

### 2.2. Data Collection

The current analysis used the dataset released in January 2018 (dataset: jecs-an-20180131), and October 2019 (dataset: jecs-ta-20190930), which comprised three types of information: (1) a self-reported questionnaire completed around the first trimester, which included basic maternal information and a food frequency questionnaire (FFQ) during the first trimester; (2) a self-reported questionnaire completed during the second/third trimester, which included socioeconomic data, such as maternal education and household income; and (3) obstetric outcomes and maternal medical background data, retrieved from the medical records of a cooperating health care provider. In the present study, we excluded participants with insufficient data and multiple pregnancies.

### 2.3. Determination of Preconception Se Intake

Preconception Se intake was determined using the FFQ, which was completed during the first trimester. Participants were asked how often they consumed various types of food from 1 year before pregnancy to their first trimester. These data were used to indicate dietary patterns during the preconception period. A standard portion size was specified for each item of the FFQ. Response options for the intake frequency ranged from almost never to ≥7 times/day for foods such as shrimps, seaweed, and several types of meat, including chicken, beef, and pork, and from almost never to ≥10 glasses/day for beverages such as tomato juice. The intake frequencies were then multiplied by the specified portion size. The nutrient content of each food was obtained from the standard table of food composition in Japan, 5th revision [24], and the daily intake of Se was estimated by summing the contents from all the food items after multiplying by the frequency of consumption. The FFQ has been validated as a self-administrated dietary questionnaire for the Japanese general population as it is structured according to the eating habits of the Japanese population [25]. 

### 2.4. Diagnosis of GDM in Japan

In Japanese medical practice, glucose-tolerance screening and testing for GDM is performed as a universal screening for every woman according to the protocols of the Obstetrics Society or Diabetes Society of Japan, depending on the local institution, using a two-step protocol [26,27]. All pregnant women participating in JECS underwent the screening procedure for GDM diagnosis during both early and late pregnancy. In Japan, glucose-tolerance screening and testing for GDM is performed universally for every pregnant woman, according to the protocols recommended by the Obstetrics Society or Diabetes Society of Japan and depending on the local obstetrics institution, as a two-step protocol during both first and second/third trimester. In short, the first step is the screening of random blood glucose (RBG) levels or a fasting 1 h 50 g oral glucose challenge test (GCT) levels during the first trimester. If the screening is positive, the pregnant women undergo a 75 g oral glucose tolerance test (OGTT) and are confirmed with GDM. If the first trimester screening is negative, a second screening using either RBG or a fasting 1 h 50 g GCT is performed during the second/third trimester. An RBG ≥95 mg/dL or a GCT result of >140 mg/dL is considered a positive screening result. In case of a positive screening result, 75 g OGTT (cutoff values of ≥92 mg/dL for fasting plasma glucose, ≥180 mg/dL for blood glucose at 1 h, and ≥153 mg/dL for plasma glucose at 2 h) is conducted. GDM is diagnosed if at least one of three glycemic levels are above thresholds during OGTT (fasting plasma glucose, plasma glucose at 1 h, and plasma glucose at 2 h).

As Japan has a unique GDM screening system conducted in two pregnancy periods (the early and mid-trimesters), we further categorized GDM into early-onset (Eo) GDM (diagnosed before 24 weeks) and late-onset (Lo) GDM (diagnosed after 24 weeks) (unpublished data). Participants with DM before pregnancy, maternal serum glycated hemoglobin levels ≥6.5% in the first trimester, and any steroid use during pregnancy were excluded from the present study.

### 2.5. Measurement of 8-OHdG Levels

Urine 8-OHdG levels (ng/mL) were estimated during the second/third trimester by liquid-chromatography-tandem mass spectrometry. Urinary creatinine level was determined as a proportion of the 8-OHdG excreted in the urine [28].

### 2.6. Other Obstetric Outcomes and Confounding Factors

Other obstetric outcomes obtained from the M0 data included the following: preterm birth, defined as delivery prior to 37 gestational weeks, and low birth weight (LBW), defined as a neonatal weight of <2500 g at the time of delivery.

The following parameters were considered to be confounding factors: maternal age, body mass index (BMI) before pregnancy, maternal smoking status, parity, chronic hypertension before pregnancy, and use of assisted reproductive technology (ART). Participants were categorized into four age groups: <20, 20–29, 30–39, and ≥40 years. The BMI was calculated according to the World Health Organization standards (body weight [kg]/height^2^ [m^2^]). We further categorized the participants into three groups according to their BMI: <18.5, 18.5–25.0, and >25.0 kg/m^2^. A self-reported questionnaire during the first and second trimesters provided information on their smoking history: “Never,” “Previously did, but quit before realizing the current pregnancy”, “Previously did, but quit after realizing the current pregnancy”, and “Currently smoking.” Women who reported to be “Currently smoking” were considered as smokers (smoking category); otherwise, they were considered as nonsmokers. Participants were also categorized based on the number of previous deliveries: 0 (primipara) and 1 or more (multipara). The method of conception was categorized as natural or ART-related, with ART defined as conception after in vitro fertilization and/or intracytoplasmic sperm injection, or cryopreserved, frozen, or blastocyst embryo transfers [29].

### 2.7. Statistical Analyses

The participants were categorized into Se intake quintiles (Q1 had the lowest, and Q5 had the highest Se intake). Maternal characteristics were summarized according to the quintile group. Kruskal–Wallis (or one-way analysis of variance, ANOVA) and chi-square tests were used to compare continuous and categorical variables, respectively. To compare the urine 8-OHdG concentration among the five groups, Tukey’s test was conducted as a post hoc test. The adjusted odds ratios (aORs) and 95% confidence intervals (CIs) for GDM, Eo-GDM, and Lo-GDM were calculated using multiple logistic regression modelling, with maternal age, BMI, maternal smoking status, parity, chronic hypertension, and ART pregnancy as confounders. This was accomplished by using dummy variables for categorical variables with more than three categories. Statistical analyses were performed using IBM SPSS statistics, version 26 (IBM Corp., Armonk, NY, USA). A *p*-value of <0.05 indicated statistical significance.

## 3. Results

**Figure 1:**
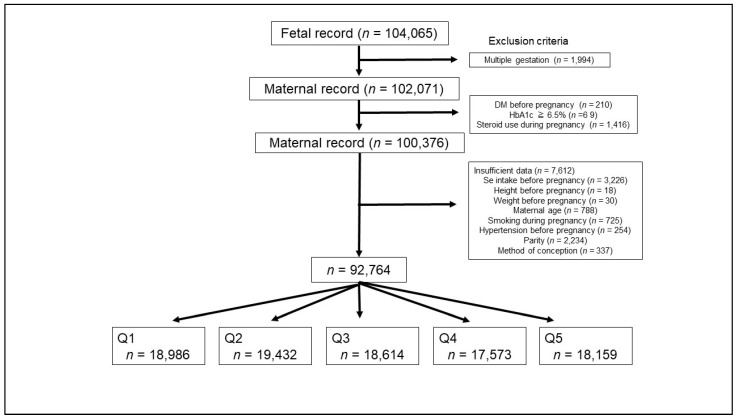
Participant selection flowchart.

After applying the inclusion and exclusion criteria, 92,764 participants were enrolled in the present analysis. These women were categorized into five groups based on the Se intake quintile (Figure 1).

### 3.1. Maternal Medical and Socioeconomic Background and Obstetric Outcomes

Table 1 summarizes the maternal medical background parameters and obstetric outcomes according to the preconception Se intake quintile. The median (interquartile range) preconception Se intake (μg/day) of each group from Q1 to Q5 was 27 (21–30), 39 (36–42), 50 (47–52), 62 (59–66), and 89 (78–111) μg/day, respectively.

There were significant differences in the categories of maternal age (*p* < 0.001), BMI before pregnancy (*p* < 0.001), smoking during pregnancy (*p* < 0.001), primipara status (*p* < 0.001), chronic hypertension (*p* = 0.023), and ART pregnancy (*p* < 0.001) among the five groups. The mean maternal age was the highest in the Q5 group (31.9 [5.0] years) and the lowest in the Q1 group (29.8 [5.3] years). Actually, the large majority of study participants had BMI between 18.5 and 25 (70%), BMI > 25 and a BMI < 18.5 were mostly seen in the Q1 group. The ratio of both smoking during pregnancy and primipara was the highest in the Q1 group. The ratio of ART pregnancy was the lowest in the Q1 group. With regard to obstetric outcomes, there were significant differences in the occurrence of GDM (*p* = 0.031), Eo-GDM (*p* = 0.043), and LBW < 2500 g (*p* = 0.005). The incidence of GDM and Eo-GDM was the highest in the Q5 group (2.9 and 0.9%, respectively).

### 3.2. Association between Preconception Se Intake and Urine 8-OHdG Levels during Early Trimester

Table 2 shows the mean value and standard error of the urine 8-OHdG levels in the five groups. There was a significant difference in the 8-OHdG levels among the five groups by one-way ANOVA. The post hoc test showed that there was a significant difference in the 8-OHdG levels (mean ng/mL [SE]) between Q1 and Q2 (2.13 [0.01] vs. 2.04 [0.01], *p* < 0.001), Q1 and Q3 (2.13 [0.01] vs. 2.04 [0.01], *p* < 0.001), Q1 and Q4 (2.13 [0.01] vs. 2.03 [0.01], *p* < 0.001), and Q1 and Q5 (2.13 [0.01] vs. 2.06 [0.01], *p* < 0.001).

### 3.3. Preconception Se intake and Risk for GDM

Table 3 summarizes the associations between preconception Se intake and the prevalence of GDM, Eo-GDM, and Lo-GDM. Using the Q3 group (the middle Se intake group) as the reference, multiple logistic regression showed that group Q1 (the lowest Se intake group) had an increased risk for Lo-GDM (aOR: 1.19, 95% CI: 1.01–1.41). Group Q5 (the highest Se intake group) had an increased risk for GDM (aOR: 1.15, 95% CI: 1.01–1.30). The Q5 group also had increased risk for both Eo- and Lo-GDM in the univariate analysis (OR: 1.34, 95% CI: 1.06–1.69 and OR: 1.21, 95% CI: 1.02–1.42, respectively). However, the risk was modified by logistic regression analysis (aOR: 1.24, 95% CI: 0.98–1.56 and OR: 1.15, 95% CI: 0.98–1.36, respectively).

## 4. Discussion

Using data from a large Japanese birth cohort study, participants were classified into five groups based on their daily Se intake from 1 year before pregnancy to their first trimester. Women in the lowest Se intake group were more likely to be of younger maternal age, primipara, have a high and low BMI, and smoke during pregnancy. The lowest Se intake was also associated with oxidative DNA damage. Using the middle Se intake group (Q3) as the reference, the highest Se intake group (Q5) had an increased risk of GDM occurrence. When we further categorized GDM into Eo- and Lo-GDM, the lowest Se intake group (Q1) had an increased risk of Lo-GDM occurrence.

In contrast to other micronutrients, the effect of Se deficiency or toxicity depends on the geographic factor. For example, deficient Se intake is associated with Keshan disease, a cardiomyopathy that is frequently fatal, named after the area in the extreme northeast of China where it was endemic [30]. In contrast, toxic Se concentrations also have harmful effects, such as hair and nail loss, poor dental health, garlic breath, nervous system disorders, and paralysis [31]. Previous case-control studies reported that high Se levels were associated with reduced DM prevalence [32,33]. Conversely, the large US National Health and Nutrition Examination Survey reported that high serum Se concentration was associated with an increased risk for DM [34]. Reduced serum Se concentration has also been reported as a risk for type 2 DM. Roman et al. reported that the mean Se concentration among Italian individuals with type 2 DM was 78 µg/L, which was significantly lower than that in individuals without type 2 DM (85 µg/L) [35].

Consistent with the previous conflicting evidence, our study showed a nonlinear association between preconception Se intake and glucose intolerance (i.e., a higher preconception Se intake is more likely to be harmful than advantageous). The reason why lower daily Se intake is thought to be associated with higher maternal oxidative stress levels is because Se is thought to have an antioxidant effect [36,37]. Therefore, the deficiency in daily Se intake, which indicates reduced antioxidant function, could be a potential risk for glucose intolerance. Our result of higher urine 8-OHdG levels in the lowest daily Se intake group supports this theory.

The increased risk for GDM in the highest Se intake group may be explained by the effect of increased Se levels on insulin signaling. Recent evidence indicates a negative effect of increased SP on insulin resistance and insulin secretion. Animal experiments revealed that injection of human SP at a concentration that increases SP concentration in diabetic patients inhibited insulin transduction in normal mice and induced hyperglycemia or insulin resistance [9,10]. The other mechanism by which excess Se intake increases the risk of GDM is that the binding of insulin to its receptor activates a burst of hydrogen peroxide that acts as a second messenger via the insulin signaling cascade [38]. The increased activity of SP, such as GP, which removes hydrogen peroxide, might interfere with insulin signaling [39]. For example, transgenic mice overexpressing GP developed insulin resistance and hyperglycemia [37]. Additionally, in pregnant women, increased erythrocyte GP activity has been associated with mild insulin resistance [40].

Recently, there is growing interest in preconception health, as preconception is a crucial period for influencing not only pregnancy outcomes but also the long-term future health of the mother and child. Preconception diet counseling could assist in providing the motivation to alter food-intake behavior during pregnancy [41]. Foods with high Se content are seafood and shellfish, and the Se content of plant foods and livestock products varies depending on the Se content in the soil and feed, respectively. Dietary Se intake also varies across regions. For example, the mean daily Se intake is 40 μg/day in Europe and 93 μg/day in the USA [19]. Japanese people consume a lot of seafood. Therefore, adult Se intake is estimated to reach an average of approximately 100 µg/day in Japan [42]. In our study, the median (interquartile range) Se intake was 50 (47–52) µg/day in the Q3 group (median Se intake group), and 27 (21–30) and 89 (78–111) µg/day in the Q1 (lowest Se intake group with increased risk for Lo-GDM) and Q5 groups (highest Se intake group with increased risk for GDM), respectively. The Ministry of Health, Labor, and Welfare in Japan set the estimated average requirement, recommended dietary allowance, and tolerable upper intake level of Se among reproductive age women at 20, 25, and 350 µg/day, respectively [42]. Our study results suggest that the estimated average requirement and recommended dietary allowance for reproductive-age women need to be increased, while the tolerable upper intake level needs to be decreased to prevent GDM.

To the best of our knowledge, this study is the first to examine the association between preconception Se intake and the occurrence of GDM, as well as the association between daily preconception Se intake and degree of oxidative DNA damage. Therefore, a strength of the present study is that it used data from the first large-scale birth cohort study conducted in Japan by the Japanese government with meticulous attention to data collection and is therefore considered to be representative of the general pregnant population in Japan [43]. Randomized control intervention trials are frequently considered the best study design; however, it is impossible to conduct a long-term control trial that examines the overall diet intake as the exposure. Although the present results were not derived from a randomized control study, the large-scale nature of this cohort study allowed evaluation of the associations between GDM and preconception exposures.

The present study also has potential limitations. First, although we accounted for numerous confounding factors, covering large portions of the questionnaires, other unknown factors may affect the occurrence of GDM. Second, we are not aware of the glycemic condition, such as the result of RBG, a fasting 50 g GCT, or 75 g OGTT, that might affect each obstetric outcome [44]. Third, Se intake was determined using a self-reported questionnaire and not by a nutritionist, and this could affect the reliability of the result. Instead, we assessed the urine 8-OHdG level and found that lower daily Se intake, which indicates reduced antioxidant defense, is associated with higher urine 8-OHdG levels, indicating higher oxidative stress levels. Fourth, the FFQ includes dietary information for 1 year before pregnancy but was completed in the first trimester; therefore, a recall bias related to morning sickness may exist. Finally, since the FFQ of the JECS referred to Japanese food customs and Japanese women were targeted, the study results may not be generalizable to other ethnicities.

## 5. Conclusions

The present study indicates a U-shaped association between preconception Se intake and increased risk for GDM. Therefore, maintaining appropriate Se intake before pregnancy is a potential prevention strategy against GDM. We hope that the present study provides the upper and lower limits of Se intake for women of reproductive age to improve both the maternal and offspring’s short- and long-term health. Further studies examining the correlation between intake of antioxidant diet before pregnancy and objective serum or urine biomarkers may prove a causal relationship between daily Se intake and the occurrence of GDM.

## Figures and Tables

**Table 1:** Maternal basic background and obstetric outcomes according to the quintile for preconception Se intake.

	Quintile for Se Intake					
	Q1 (Low)	Q2	Q3	Q4	Q5 (High)	
Variable	*n* = 18,986	*n* = 19,432	*n* = 18,614	*n* = 17,573	*n* = 18,159	*p*-Value
Maternal medical background						
Preconception Se intake, μg/day median (interquartile range)	27 (21–30)	39 (36–42)	50 (47–52)	62 (59–66)	89 (78–111)	<0.001 ^a^
Maternal age, mean years (SD)	29.8 (5.3)	31.1 (5.0)	31.5 (4.9)	31.8 (4.8)	31.9 (5.0)	<0.001 ^b^
Maternal age category, %						
≤19	1.6	0.7	0.7	0.5	0.6	<0.001 ^c^
20–29	47.1	37.3	34.2	32	31.3	
30–39	48.2	57.5	60.3	62.5	62.4	
≥40	3.1	4.6	4.8	5.0	5.7	
BMI before pregnancy (kg/m^2^), %						
<18.5	17.1	16.0	16.2	16.0	15.4	<0.001 ^c^
18.5–25.0	70.8	73.9	73.9	74.2	73.2	
>25.0	12.1	10.1	9.9	9.8	11.4	
Smoking during pregnancy, %	6.4	4.7	4.1	3.9	5.2	<0.001 ^c^
Primipara, %	48.2	42.4	38.8	36.6	34.7	<0.001 ^c^
Hypertension before pregnancy, %	1.3	1.2	1.1	1.0	1.3	0.023 ^c^
ART, %	2.2	3.0	2.9	3.3	3.1	<0.001 ^c^
Obstetric outcomes						
GDM, %	2.5	2.7	2.4	2.6	2.9	0.031 ^b^
Eo-GDM, %	0.7	0.8	0.7	0.8	0.9	0.043 ^b^
Lo-GDM, %	1.6	1.6	1.4	1.5	1.7	0.191 ^b^
PTB < 37 wks, %	5.4	5.4	5.4	4.9	5.6	0.076 ^b^
LBW < 2500 g, %	8.8	8.8	8.3	7.9	8.7	0.005 ^b^

Abbreviations: Se: selenium, SD: standard deviation, BMI: body mass index, ART: assisted reproductive technology, GDM: gestational diabetes mellitus, Eo: early onset, Lo: Late onset, PTB: preterm birth, LBW: low birth weight, wks: weeks. ^a^ *p*-value, Kruskal–Wallis test; ^b^ *p*-value, one-way analysis of variance; ^c^ *p*-value, chi-square test.

**Table 2:** Maternal 8-hydroxy-2′-deoxyguanosine (8-OHdG) urine levels during pregnancy according to the preconception Se intake quintiles.

	Quintile for Se Intake					
	Q1 (Low)	Q2	Q3	Q4	Q5 (High)	
Variable	*n* = 18,986	*n* = 19,432	*n* = 18,614	*n* = 17,573	*n* = 18,159	*p*-Value
Urine 8 OHdG levels, ng/mL, mean (SE)	2.13 (0.01)	2.04 (0.01)	2.04 (0.01)	2.03 (0.01)	2.06 (0.01)	<0.001 ^a^

Abbreviations: Se: selenium, 8-OHdG: 8-hydroxy-2′-deoxyguanosine. ^a^ One-way analysis of variance and Tukey’s post-hoc test showed a significant difference between Q1 vs. Q2 (*p* < 0.001), Q1 vs. Q3 (*p* < 0.001), Q1 vs. Q4 (*p* < 0.001), and Q1 vs. Q5 (*p* < 0.001).

**Table 3:** Relationship between preconception Se intake and GDM.

	Quintile for Se Intake				
	Q1 (Low)	Q2	Q3	Q4	Q5 (High)
	*n* = 18,986	*n* = 19,432	*n* = 18,614	*n* = 17,573	*n* = 18,159
GDM					
OR (95% CI)	1.03 (0.90–1.17)	1.09 (0.96–1.24)	1 (Ref)	1.08 (0.95–1.23)	1.21 (1.07–1.38)
aOR (95% CI)	1.07 (0.94–1.22)	1.10 (0.97–1.25)	1 (Ref)	1.07 (0.94–1.22)	1.15 (1.01–1.30)
Eo-GDM					
OR (95% CI)	0.98 (0.77–1.25)	1.15 (0.91–1.45)	1 (Ref)	1.20 (0.95–1.53)	1.34 (1.06–1.69)
aOR (95% CI)	1.02 (0.80–1.31)	1.16 (0.92–1.47)	1 (Ref)	1.19 (0.93–1.51)	1.24 (0.98–1.56)
Lo-GDM					
OR (95% CI)	1.16 (0.98–1.37)	1.13 (0.96–1.33)	1 (Ref)	1.06 (0.89–1.26)	1.21 (1.02–1.42)
aOR (95% CI)	1.19 (1.01–1.41)	1.14 (0.96–1.34)	1 (Ref)	1.05 (0.89–1.25)	1.15 (0.98–1.36)

Abbreviations: Se: selenium, GDM: gestational diabetes mellitus, Eo: early-onset, Lo: late-onset, OR: odds ratio, aOR: adjusted odds ratio, CI: confidence interval, Ref: reference. aOR was calculated by logistic regression analysis, using maternal age (20–29 years as reference), ART pregnancy, body mass index before pregnancy (18.5–25.0 as reference), maternal smoking status, maternal education, parity, and chronic hypertension before pregnancy.

## Data Availability

Data are unsuitable for public deposition due to ethical restrictions and legal framework of Japan. It is prohibited by the Act on the Protection of Personal Information (Act No. 57 of 30 May 2003, amendment on 9 September 2015) to publicly deposit the data containing personal information. Ethical Guidelines for Medical and Health Research Involving Human Subjects enforced by the Japan Ministry of Education, Culture, Sports, Science, and Technology and the Ministry of Health, Labor, and Welfare also restrict the open sharing of the epidemiologic data. All inquiries about access to data should be sent to jecs-en@nies.go.jp. The person responsible for handling enquiries sent to this e-mail address is Shoji F. Nakayama, JECS Programme Office, National Institute for Environmental Studies.
